# New vectors that are early feeders for *Plasmodium knowlesi* and other simian malaria parasites in Sarawak, Malaysian Borneo

**DOI:** 10.1038/s41598-021-86107-3

**Published:** 2021-04-08

**Authors:** Joshua Xin De Ang, Khatijah Yaman, Khamisah Abdul Kadir, Asmad Matusop, Balbir Singh

**Affiliations:** 1 Malaria Research Centre, Faculty of Medicine and Health Sciences, Universiti Malaysia Sarawak, Kuching, Sarawak Malaysia; 2 Sarawak Department of Health, Kuching, Sarawak Malaysia

**Keywords:** Malaria, Phylogenetics, Epidemiology

## Abstract

*Plasmodium knowlesi* is the main cause of malaria in Sarawak, where studies on vectors of *P. knowlesi* have been conducted in only two districts. *Anopheles balabacensis* and *An. donaldi* were incriminated as vectors in Lawas and *An. latens* in Kapit. We studied a third location in Sarawak, Betong, where of 2169 mosquitoes collected over 36 days using human-landing catches, 169 (7.8%) were *Anopheles *spp. PCR and phylogenetic analyses identified *P. knowlesi* and/or *P. cynomolgi, P. fieldi, P. inui*, *P. coatneyi* and possibly novel *Plasmodium spp.* in salivary glands of *An. latens* and *An. introlatus* from the Leucosphyrus Group and in *An. collessi* and *An. roperi* from the Umbrosus Group. Phylogenetic analyses of cytochrome oxidase subunit I sequences indicated three *P. knowlesi*-positive *An. introlatus* had been misidentified morphologically as *An. latens,* while *An. collessi* and *An. roperi* could not be delineated using the region sequenced. Almost all vectors from the Leucosphyrus Group were biting after 1800 h but those belonging to the Umbrosus Group were also biting between 0700 and 1100 h. Our study incriminated new vectors of knowlesi malaria in Sarawak and underscores the importance of including entomological studies during the daytime to obtain a comprehensive understanding of the transmission dynamics of malaria.

## Introduction

*Plasmodium knowlesi,* primarily a simian malaria parasite*,* was reported to have caused a large number of human malaria infections in 2004 in the Kapit Division of Sarawak, Malaysian Borneo^1,2^. Subsequently knowlesi malaria cases have been reported throughout Southeast Asia and they continue to be a public health concern, particularly in Malaysia. From 2017 to 2019, a total of 10,968 knowlesi malaria cases were reported in Malaysia, with 87% occurring in the Malaysian Borneo states of Sabah and Sarawak (unpublished data, Ministry of Health Malaysia)^3^. Studies investigating the epidemiology of the parasite and the identity of its mosquito vectors have seen significant progress since the initial report in 2004. For example, long-tailed and pig-tailed macaques were identified as the reservoir hosts for *P. knowlesi* and other simian malaria parasites (*P. coatneyi, P. cynomolgi, P. fieldi,* and *P. inui*) in Sarawak^4^. Furthermore, phylogenetic analyses of the mitochondrial genomes have shown previously unreported species of *Plasmodium* in long-tailed macaques in Sarawak; *P. inui-*like parasites and *P. simiovale*^5^. In addition, microsatellite typing of *P. knowlesi* derived from humans and monkeys and whole genome sequencing of clinical samples have demonstrated the existence of at least three *P. knowlesi* subpopulations, two of which occur in Malaysian Borneo and the others in Peninsular Malaysia^6,7^. In terms of entomological studies, several species from the Leucosphyrus Group, two species from the Barbirostris Group, and a species from the Sundaicus Complex have been implicated as vectors of *P. knowlesi* in India (*An. sundaicus*), Vietnam (*An. dirus*), Peninsular Malaysia (*An. hackeri*, *An. cracens* and *An. introlatus*), Sabah (*An. balabacensis* and *An. donaldi*), and Sarawak (*An. latens, An. balabacensis,* and *An. donaldi*)^8–18^.

An effective vector control strategy is key to successful malaria prevention and control, but it will have to be devised based on accurate understanding of the identity and bionomics of the vectors at the location of transmission. This was previously demonstrated in Vietnam where a non-vector (*An. varuna*) was misidentified as the vector *An. minimus* and subsequently wrongly targeted for vector control^19^, causing the ineffective use of valuable and limited resources. Misidentification however, is relatively common in vector studies and especially where vectors comprise species complexes which are difficult to distinguish morphologically^20–23^. For this reason, molecular methods based on the cytochrome oxidase subunits I and II (COI and COII), second internal transcribed spacer (ITS2) within the ribosomal DNA, and NADH dehydrogenase sub-unit six (ND6) have been developed as a complementary tool to improve accuracy in identification of vectors^20,22,24–32^.

There remain gaps in knowledge regarding the vectors involved in the transmission of *P. knowlesi* and other simian malaria parasites in Malaysian Borneo. Most of the entomological studies were conducted following a similar pattern where mosquitoes were first collected in the field for a 6- or 12-h duration beginning at 1800 h or 1900 h. The mosquitoes were morphologically identified, in certain studies they were dissected, and DNA that was extracted was then analysed by PCR assays. These assays were designed to only detect the five simian malaria parasites initially described in macaques^4^. Consequently, it is unknown if other *Plasmodium* species could be transmitted by these mosquitoes. It is also not known if other species of vectors could be responsible for malaria transmission if a different collection period was adopted. Furthermore, previous studies have been carried out only in the Kapit and Lawas Districts of Sarawak, so entomological studies need to be conducted in many other localities in this large state which has a land area of 124,450 km^2^. Therefore, the aims of the current study were to identify vector(s) and the species of *Plasmodium* they transmit in the Betong District of Sarawak and to determine whether there are any vectors that feed earlier in the day.

## Results

### Species composition of mosquitoes

**Figure 1:**
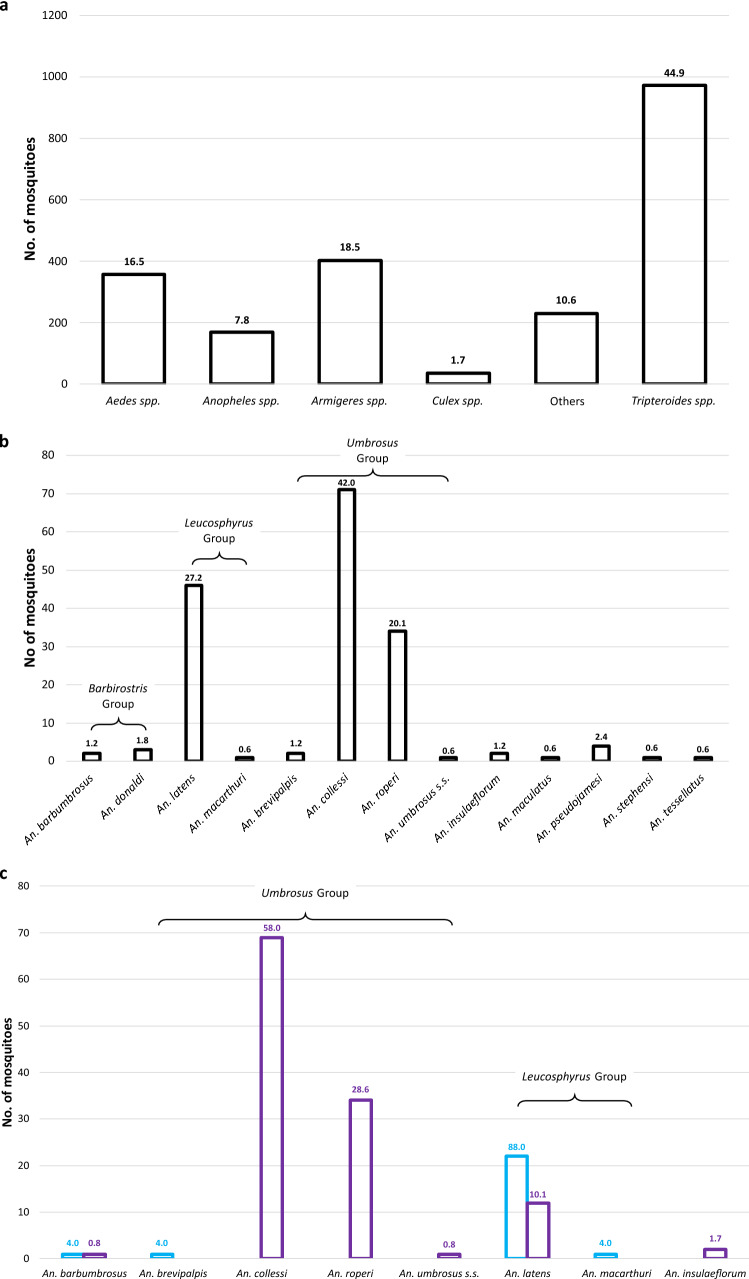
Bar chart representing the number of mosquitoes, according to morphological identification, collected in this study. Percentages of each species are shown above each bar. (**a**) All mosquitoes from all sites. (**b**) *Anopheles* mosquitoes from all sites. (**c**) *Anopheles* mosquitoes collected from sites B4 (blue) and SM (purple).

A total of 2169 mosquitoes were collected from all the collection sites from April 2015 to November 2016. Only 169 (7.8%) were identified morphologically as anophelines while the rest were culicines (92.2%) from other genera (Fig. 1a). Fourteen different species were identified and members of the Leucosphyrus and Umbrosus Groups were found to comprise more than 90% of the total *Anopheles* mosquitoes collected (Fig. 1b). *Anopheles collessi* and *An. roperi* were found to be the predominant species in site SM and both species were not collected during the ten collections in site B4 (Fig. 1c). *An. latens* was found to be the most common species found in site B4 and it was also present in site SM.

### Identification of* Plasmodium* species

Only 6 out of 85 cases of malaria at Betong District in the 2 years preceding the current study were due to infections with human malaria parasites and 79 were caused by *P. knowlesi*. Since all 6 were infections acquired from overseas, we did not include PCR primers which could specifically detect human malaria parasites in our PCR screening. The PCR assays showed that *An. latens* is the vector of *P. cynomolgi, P. fieldi, P. inui,* and *P. knowlesi* while two members of the Umbrosus Goup had sporozoites of *P. coatneyi, P. cynomolgi*, and *P. knowlesi* (Table 1). *Plasmodium inui* and *P. fieldi* were detected only in *An. latens* and not in the other *Plasmodium*-positive mosquitoes. Two *An. latens* and two *An. roperi* were also each found to have two or more species of simian malaria parasites in their salivary glands. Two *An. roperi* and one *An. collessi* infected with simian malaria parasites were caught landing on humans during the morning collections. The 11 *Anopheles* mosquitoes which were found to have sporozoites of one or more simian malaria parasites comprised 28.2% of the 39 samples which were *Plasmodium*-positive by nested PCR assays.

**Table 1:** Nested PCR results conducted on genomic DNA extracted from mosquito salivary glands.

Species series/group	Species as identified morphologically	No. collected	No. of positive anophelines		Sample ID (collection site)
			Genus-positive	Species-positive	
Leucosphyrus group	*An. latens*	46	15	1: *Pcy* + *Pfld* + *Pin* + *Pk* 1: *Pcy* + *Pfld* + *Pin* 1: *Pin* 3: *Pk*	B0362 (B4) B1056 (RL1) B1057 (RL1) B1170 (B4), B1283 (B4), B1444 (SM)
	*An. macarthuri*	1	0	–	–
Umbrosus group	*An. brevipalpis*	2	0	–	–
	*An. collessi*	71	11	2: *Pk*	B0870^a^ (SM), B1388 (SM)
	*An. roperi*	34	13	1: *Pct* + *Pcy* + *Pk* 1: *Pct* + *Pk* 1: *Pk*	B2000^a^ (SM) B1991 (SM) B1999^a^ (SM)
	*An. umbrosus* s.s	1	0	–	–
Aitkenii group	*An. insulaeflorum*	2	0	–	–
Barbirostris group	*An. barbumbrosus*	2	0	–	–
	*An. donaldi*	3	0	–	–
Jamesii group	*An. pseudojamesi*	4	0	–	–
Maculatus group	*An. maculatus*	1	0	–	–
Neocellia series	*An. stephensi*	1	0	–	–
Tessellatus group	*An. tessellatus*	1	0	–	–
Total		169	39	11	–

^a^Samples which were collected in the morning (0600–1100 h).

**Figure 2:**
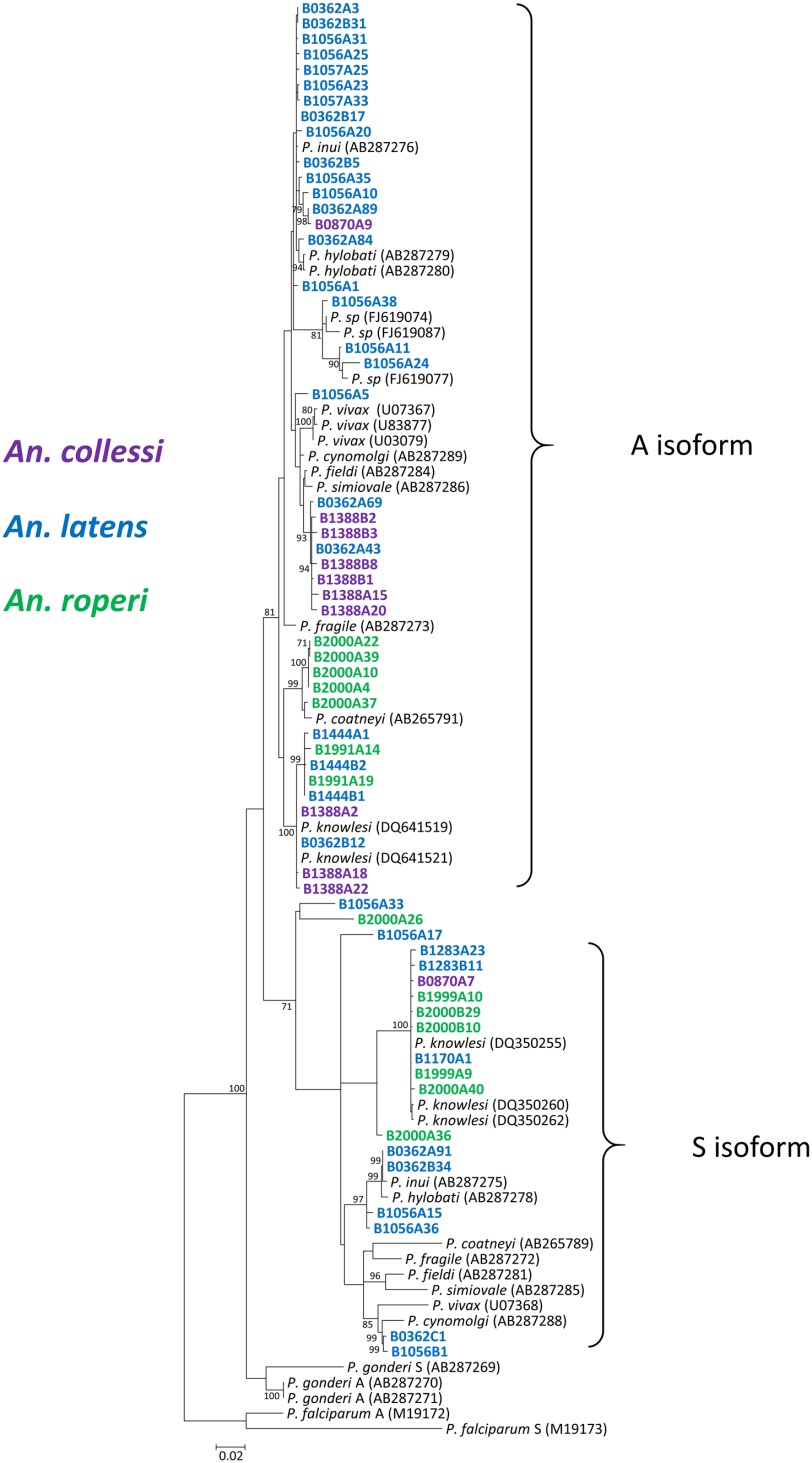
Phylogenetic tree based on the *Plasmodium* SSUrRNA genes using the ML method. The analysis was based in the Tamura-3 + G + I substitution model. GenBank accession numbers are in brackets and only bootstrap values > 70% are shown at the nodes. Sequences derived from mosquitoes morphologically identified as *An. collessi*, *An. latens* and *An. roperi* are labelled in purple, blue and green respectively. The tree was generated with MEGA 7.0.21 (https://www.megasoftware.net/)^57^.

Following PCR assays, the *Plasmodium* SSUrRNA genes were sequenced from these 11 samples for phylogenetic analysis. For six of these samples, *Plasmodium* species identified by both the PCR and ML tree were consistent but discrepancies between the two methods were observed for the remaining five samples. For example, only *P. knowlesi* was detected by PCR assays in samples B0870 and B1388, but *P. inui*- and *P. fieldi*-like SSUrDNA sequences, respectively, were derived from these mosquitoes. Furthermore, *P. coatneyi* and *P. cynomolgi* SSUrDNA sequences were not derived from samples BB1991 and B2000, respectively, even though these parasites were detected in these samples via nested PCR assays (Fig. 2). Multiple sequences derived from samples B0362, B1056, and B1057 which were identified by PCR assays as *P. fieldi* and/or *P. inui* were found to be closely related to those two species but the bootstrap values of the nodes were below 70%*.* In addition, a number of sequences were also derived from samples B1056 and B2000 which were genetically distinct from the referral sequences. Consistent with the PCR results, *P. coatneyi* sequences were only isolated from *An. roperi*.

### Phylogenetic analysis of partial COI gene sequences

**Figure 3:**
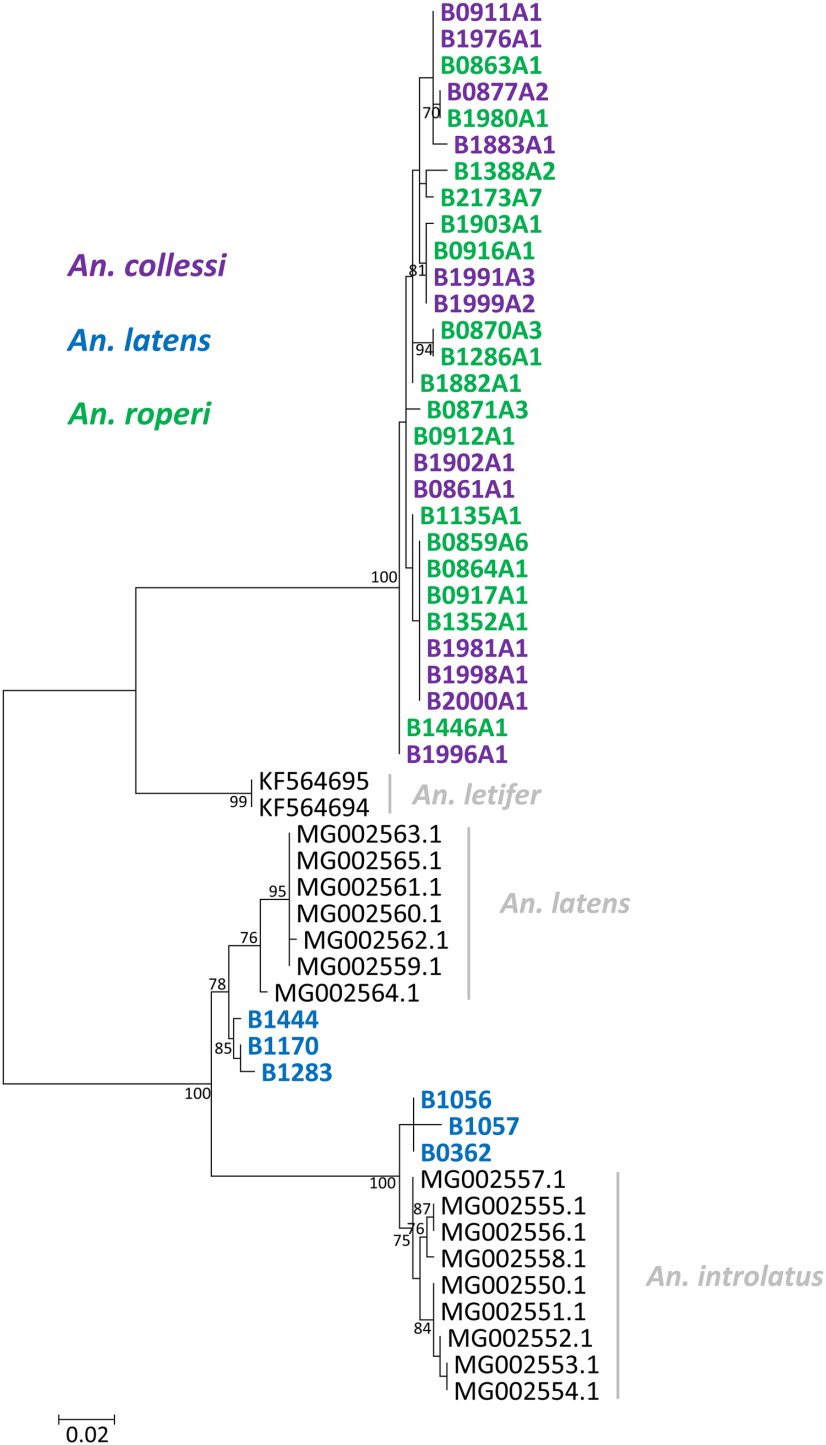
Phylogenetic tree based on the *Anopheles* COI gene using the ML method. The analysis was based in the Tamura-3 + G substitution model. Accession numbers of *An. introlatus*, *An. latens*, and *An. letifer* sequences obtained from GenBank are labelled in black while those derived from mosquitoes morphologically identified as *An. collesi*, *An. latens* and *An. roperi* are labelled in purple, blue and green respectively. The tree was generated with MEGA 7.0.21 (https://www.megasoftware.net/)^57^.

In order to confirm morphological identification of the vectors, part of the COI gene was sequenced from the 11 *Anopheles* mosquitoes that were positive for simian malaria parasites by PCR assays (Table 1). In addition to these samples, 24 more *An. collessi* and *An. roperi* were also subjected to sequencing due to the lack of previous molecular characterisation of mosquitoes of the Umbrosus Group. These COI sequences were subsequently submitted to Barcode of Life Database (BOLD) for identification. When the COI gene of six morphologically identified *An. latens* were submitted to BOLD, three of them were identified as *An. latens* while the other three were identified as *An. introlatus,* a closely-related species within the same species complex as *An. latens*. The phylogenetic tree (Fig. 3) constructed with these sequences also supports the identification by BOLD, indicating that the *An. introlatus* (B0362, B1056, and B1283) were misidentified through taxonomic keys as *An. latens*. *Anopheles latens* were found to be infected with only *P. knowlesi* while *An. introlatus* had sporozoites of multiple simian malaria species (Table 1). None of the sequences of the members of the Umbrosus Group could be identified by BOLD. When they were aligned with the other *An. leucosphyrus* s.l. to infer phylogeny, these sequences formed a distinct monophyletic clade which is closely related to *An. letifer* (KF564694 and KF564695) collected from Singapore (Fig. 3). Mosquitoes which were morphologically identified as *An. collessi* and *An. roperi* did not form distinct clades in the ML phylogenetic tree.

### Biting behaviour of vector species

**Figure 4:**
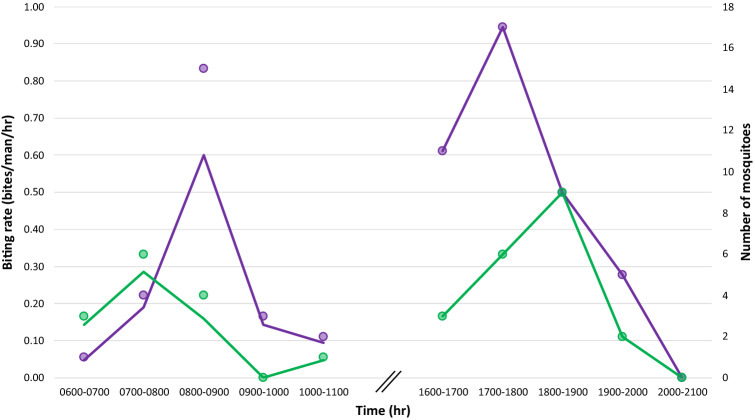
Biting behaviour (line) and the number (circle) of *An. collessi* (purple) and *An. roperi* (green) collected from site SM between 0600–1100 h and 1600–2100 h. Two circles overlap between 1800 and 1900 h.

As some of the morphologically identified *An. latens* may have been *An. introlatus,* their biting rates at specific time interval are not presented here but it is important to note that among the 46 collected from different sites, only one (B1444) from site SM started biting as early as 1600 h; the remaining 45 only started landing and feeding between 1800 and 2100 h. *Anopheles collessi* (n = 67) and *An. roperi* (n = 34) were found to bite throughout the collection period with peak biting times between 1700 and 1800 h and 1800–1900 h, respectively at site SM (Fig. 4). The only period where *An. collessi* was not landing on humans to feed was between 2000 and 2100 h while for *An. roperi*, it was from 0900 to 1000 h and 2000–2100 h. Despite being abundantly found in site SM, neither of these two genetically closely-related species were found in site B4, which was approximately 500 m from site SM.

## Discussion

With regards to the detection of *Plasmodium*, observations including a higher sporozoite rate in vectors compared to other studies^8,9,11,13–15^; discrepancy between PCR and sequencing results, and the presence of *Plasmodium* species which could not be detected with the primers specific for the five simian malaria parasites that are reported in the current study, are consistent with the observations from our previous study in Lawas, Sarawak^18^. We have suggested that the high sporozoite rates observed could be attributed to a higher sensitivity of the sampling and detection methods since we used all the salivary glands for DNA extraction and we detected *Plasmodium* with molecular methods. Furthermore, since the *Plasmodium*-specific PCR assay uses primers which can amplify both the A and S types of SSU rDNA as opposed to the species-specific primers that are specific for either the A or S type, this indicates that either the number of sporozoites were low and/or the current primers could not detect other species of *Plasmodium* present in 28 of the *Plasmodium*-positive *Anopheles* mosquitoes. *Plasmodium* infection of diverse species with varying densities in these mosquitoes could cause the other two observations. It is also possible that the high *Plasmodium* detection rate observed is due to the close proximity of sites B4 and SM to wildlife such as long-tailed macaques, which were noted near site SM during one of the early collections.

Some of the sequences generated in our study did not fall into any particular clade following phylogenetic analysis and the reasons for this could be either that the SSUrRNA gene is not a suitable marker for identification of species of *Plasmodium* or that there are species of *Plasmodium* circulating among wildlife in Betong and Lawas that have not previously been characterised^18^. It would be necessary to sequence additional genes in order to confirm that there are novel species of *Plasmodium* carried by these mosquitoes as was done recently with non-nuclear genes^5^. Phylogenetic analyses of the *Plasmodium* mitochondrial genome and apicoplast caseinolytic protease M gene revealed the existence of two subpopulations of *P.* inui-like parasites in long-tailed macaques^5^ in Sarawak and that these macaques are new hosts for *P. simiovale*. In addition, microsatellite genotyping and whole genome sequencing has shown that there are three *P. knowlesi* subpopulations present in human patients and laboratory-maintained *P. knowlesi* strains from macaques^6,33^. Sequences labelled as *P.* cf. *inui, P.* cf. *fieldi,* and *P.* sp. in Fig. 2 were all unidentified sequences derived from long-tailed macaques in the Kapit Division of Sarawak^34^. These observations suggest that sequences which did not fall into a particular clade following phylogenetic analysis in the current study possibly represent novel *Plasmodium* species. However, it is not known at this stage, if these *Plasmodium* species would pose any threat to human health.

Phylogenetic analysis revealed three *An. introlatus* had probably been misidentified through taxonomic keys as *An. latens* and it was not possible to distinguish between the samples which were morphologically identified as *An. collessi* and *An. roperi.* This inability to differentiate *An. collessi* from *An. roperi* could be due to the short lengths of the COI sequences that were used for the phylogenetic analysis. Although the primers used in the present study generated fragments of approximately 1400 bp of the COI gene following PCR amplification, only 700 nucleotides were aligned during phylogenetic analyses because longer reference sequences were not available in GenBank. If a larger fragment of the gene was used in the alignment, the phylogenetic analysis may have had a greater capacity in determining the identity of the species of these mosquitoes. It is also possible that the COI gene is not a suitable marker for distinguishing different species in certain species complexes. This was demonstrated by Carter et al.^35^ in their recent attempt to use both the ITS2 and COI sequences to identify mosquito species in Ethiopia. They successfully distinguished two distantly related species, *An. arabiensis* and *An. pretoriensis* by using ITS2 but not with COI sequences. In a separate study conducted in South Sulawesi, *Anopheles* mosquitoes were identified morphologically to their genera and an approximately 700 nucleotide fragment of the COI gene was sequenced from each individual (n = 392)^36^. The sequences were aligned and were found to have separated into nine different contigs, suggesting the presence of nine species/species groups among the samples collected. By comparing the consensus sequences of the contigs to GenBank and BOLD, the identities of seven groups were determined while each of the remaining two groups had high sequence similarities (> 95%) to two distantly related species. This further demonstrates that parts of the COI gene of distantly related species could have high sequence similarity and that the gene could not be used by itself to reliably infer phylogeny. No other DNA barcodes (e.g. ITS2, COII, etc.) were sequenced for the *An. collessi* and *An. roperi* in the present study however, as sequences in the Umbrosus Group were not available in GenBank for use as reference sequences. Nevertheless, our data indicates that *An. latens*, *An. introlatus*, *An. collessi,* and *An. roperi* are vectors of *P. knowlesi* and/or other simian malaria parasites in the Betong District of Sarawak.

We have incriminated *An. roperi* and *An. collessi*, mosquitoes of the Umbrosus Group, as novel vectors of *P. knowlesi* and other simian malaria parasites in Malaysian Borneo. From the limited studies that have been undertaken, both these species have been found in several regions in Asia: *An. collessi*^37,38^ in Peninsular Malaysia, Malaysian Borneo, and Brunei, and *An. roperi*^39^ in Peninsular Malaysia, Malaysian Borneo, Thailand, Cambodia, and the Nicobar Island of India. There is a need for further studies on the geographic distribution of these vectors in order to provide specific guidelines for vector control strategies^40–42^. While *P. knowlesi* has been previously detected in the salivary glands and midguts of *An. latens* and *An. introlatus*^12,15^, respectively, this is the first time the parasite was identified in the salivary glands of *An. introlatus, An. collessi* and *An. roperi.* This is also the first time members of the Umbrosus Group are incriminated as vectors for *P. knowlesi*. Previous studies have indicated the role of the Umbrosus Group in the transmission of human and non-human malaria parasites in Malaysia. In Peninsular Malaysia in 1918, Barber^43^ had noticed "in some specimens of *An. umbrosus* s.l. the sporozoites appeared abnormally thick and short, and in other specimens they were apparently normal" while Hodgkin in 1956^44^ suspected some of the *An. umbrosus* s.l. might have been infected with monkey malaria. *Plasmodium traguli*, the malaria parasite of the mouse deer (*Tragulus kanchil*), was able to develop into sporozoites in *Anopheles umbrosus* s.s., *An. letifer, An. baezai, and An. roperi* that had been bloodfed using infected mouse deer^45^. Two other investigations have also reported the presence of oocysts and/or sporozoites (parasite identity was not mentioned) from dissected *An. baezai, An. collessi, An. letifer, An. separatus, An. roperi,* and *An. umbrosus* caught in nature^41,46^. In Sarawak, the salivary glands of *An. letifer* have been found to be infected with what the authors described as malaria parasites of non-human and human origin in Baram in the 1950s and in Miri in 1997, respectively, but they did not provide descriptions of the morphological differences between these sporozoites^47,48^. Taken together, these studies show that some members of the Umbrosus Group are able to transmit simian and other malaria parasites, although the exact identity of the species of *Plasmodium* was not stated in certain studies.

Several members of the Leucosphyrus Group (*An. cracens, An. latens, An. introlatus,* and *An. balabacensis* in Malaysia; *An. dirus* in Vietnam) and *An. donaldi* (Malaysia) have previously been incriminated in the transmission of *P. knowlesi* and other simian malaria parasites in Southeast Asia^8,9,11–16,18,49^. In all the studies where peak biting times were described, *An. balabacensis*^8,11,49^*, An. cracens*^13,14^*, An. donaldi*^11^*,* and *An. introlatus*^12^ were reported to be mostly landing on humans between 1800 and 2100 h but were already biting within the first hour of the start of the collection period in the evenings. *Anopheles latens* was reported to have peak biting activity between 1900–2000 h and 0100–0200 h in the forest and farm settings, respectively, in the Kapit district of Sarawak, Malaysian Borneo but hourly peak biting rates were not provided^50^*.* The peak biting rates for *An. balabacensis* (approximately 0.18 bites/man/h at 1900–2000 h) and *An. donaldi* (approximately 0.35 bites/man/h at 1800–1900 h) determined from 6-h night collections in a study at Ranau and Keningau in Sabah, Malaysian Borneo^11^ appear to be lower than our peak biting rates for *An. collessi* (approximately 0.94 bites/man/h at 1700–1800 h and 0.60 at 0800–0900 h) and *An. roperi* (0.50 bites/man/h at 1800–1900 h and approximately 0.29 at 0700–0800 h). However, we only collected mosquitoes from 0600 to 1100 h and 1600–2100 h, so we cannot rule out the possibility that at other time periods, *An. collessi* and *An. roperi* may have peak biting rates greater or lower than those we observed. The other investigations for vectors of knowlesi malaria involved 12 h-long mosquito collections at night, where the peak biting times were described as bites/man/night for each month and ranged from approximately 0–13 for *An. latens* in Kapit, Sarawak^50^, approximately 1–8 for *An. cracens* in Kuala Lipis, Peninsular Malaysia^13^, and approximately 2–28 for *An. balabacensis* in Banggi Island and Kudat, Sabah^8^. It is necessary to conduct further studies involving longer durations of collection times and monthly mosquito collections lasting 3 days each for over a year before valid comparisons of peak biting rates of mosquitoes at Betong can be made with those described by other investigators. Such studies will lead to a greater understanding of the bionomics of *An. collessi* and *An. roperi*.

Previous entomological surveys for *P. knowlesi* vectors have all been conducted between dusk and dawn (for a 6- or 12-h period starting from 1800 h or 1900 h) to coincide with what was widely accepted as the period when most malaria vectors were seeking a bloodmeal^8,9,11–16,49,50^. However, that widely held belief was derived mostly from indoor collections which reported the absence of endophagic *Anopheles* mosquitoes decades ago when human-to-human malaria transmission was the prevalent mode of transmission^41,51,52^. This assumption is therefore not applicable to vectors of *P. knowlesi* where transmission predominantly occurs outdoors^2,8,9,11,13–15,50^. Among the malaria vectors in Malaysia, *An. letifer, An. umbrosus* s.s.*, An. roperi,* and *An. donaldi* have been previously shown to feed on humans in forested shade when disturbed at their resting sites between 0600 and 1200 h^41,53,54^. While the dusk-to-dawn sampling period has enabled the incrimination of *An. leucosphyrus* s.l., and more recently *An. donaldi*^11,18^*,* as vectors of knowlesi malaria, it is possible that other vectors could have been discovered if collections were also conducted during the 12-h period between dawn to dusk. With the incrimination of *An. collessi* and *An. roperi,* which have peak biting times at 0700–0900 h and 1700–1900 h in Betong, dawn-to-dusk mosquito collections need to be utilised in future studies for a better understanding of the vectors and the transmission dynamics of knowlesi malaria*.*

In conclusion, this study has incriminated two members of the Umbrosus Group (*An. collessi* and *An. roperi*) and two members of the Leucosphyrus Complex (*An. latens* and *An. introlatus*) as vectors of *P. knowlesi* and other simian malaria parasites in Betong, Sarawak, Malaysian Borneo. Phylogenetic analyses of the *Plasmodium* SSUrRNA sequences derived from the salivary glands of these mosquitoes have indicated these vectors may also transmit novel species of *Plasmodium*. This is the first time members of the Umbrosus Group, which feed on humans in the evening as well as in the morning, were shown to harbour simian malaria parasites, underscoring the importance of also conducting entomological surveys during the daytime in order to obtain a comprehensive understanding of vectors and the transmission dynamics of malaria.

## Methods

### Study sites and collection periods

**Figure 5:**
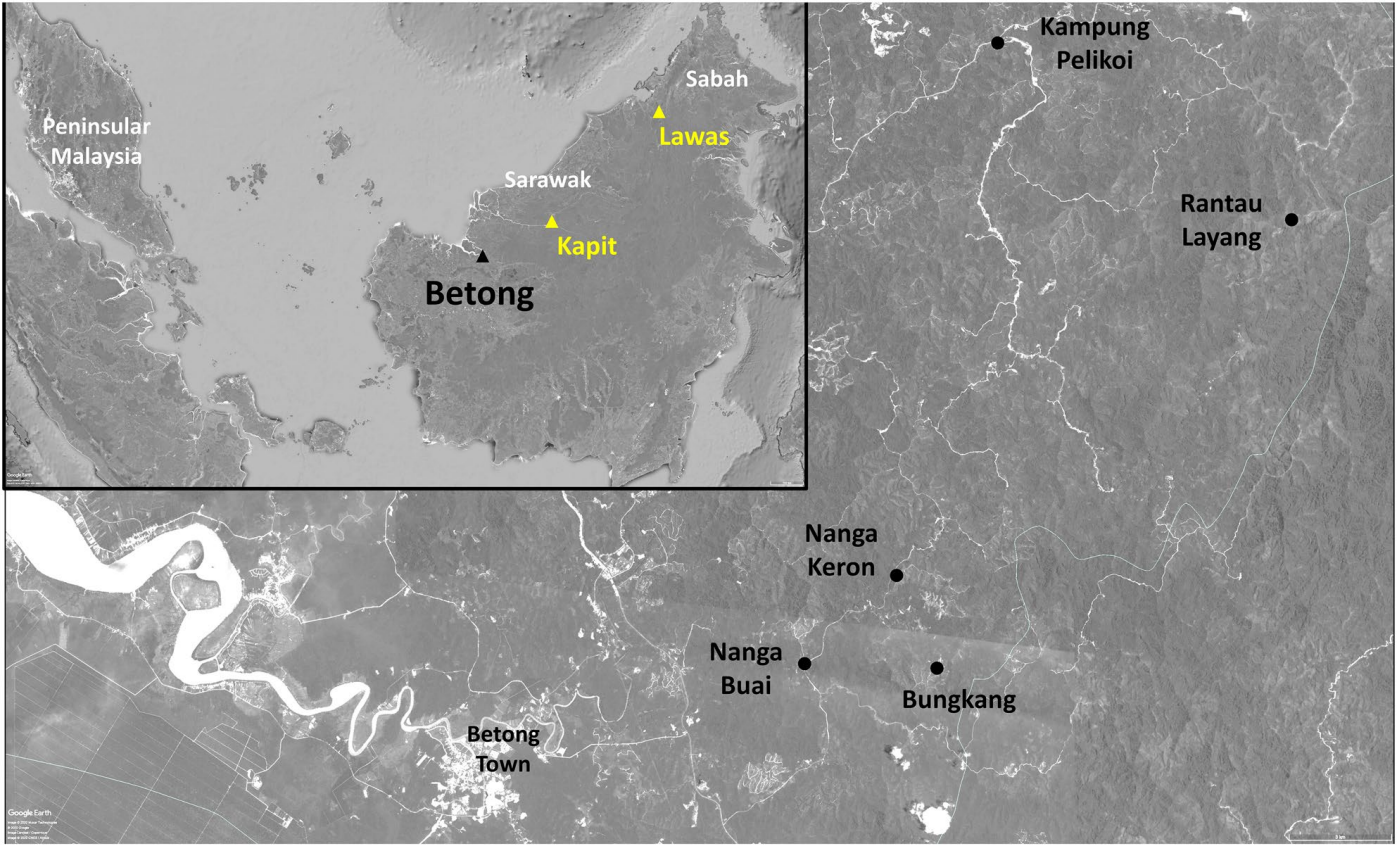
Map showing the five villages (black circles) of mosquito collection sites relative to Betong town and inset map showing where previous studies (yellow triangles) on vectors of knowlesi malaria were conducted in Sarawak. The images were obtained from Google Earth Pro 7.3.3.7786 (https://www.google.com/intl/en_uk/earth/versions/#earth-pro)^58^.

The study was carried out in the Betong District of Sarawak where 93% (79/85) of human malaria infections were caused by *P. knowlesi* in the two years preceding the start of the study in 2015. The other six were caused by human malaria parasites and all these infections had been acquired overseas (Betong Health Department, unpublished data). Mosquito collection sites were selected during the initial stage of the study based on the following criteria: (a) occurrence of *P. knowlesi* infection in the longhouse community one month prior to collection; (b) within close proximity to areas where long-tailed and/or pig-tailed macaques had been sighted and; (c) farm/hunting ground where the person infected with *P. knowlesi* had potentially acquired the infection. Mosquitoes were collected for 36 days during 8 field trips in the months of April, August, and October 2015, and in March, April, May, August, and November 2016. Although multiple collection sites near to 5 longhouses (see Supplementary Table S1 and Fig. 5) were selected in the beginning of the study, most of the local guides (previously infected with *P. knowlesi*) from the longhouses were not always available to guide the team to the collection sites. Sites B4 and SM close to the Bungkang longhouse were therefore selected as the main collection sites due to the availability of a local guide and the presence of a higher number of anophelines compared to the other sites from the initial surveys. These two sites were also the only sites where both the early (0600–1100 h) and late (1600–2100 h) collections were carried out, while in the other sampling locations only the late collections were conducted. Site B4 was a slope on a hill situated approximately 600 m southeast of the Bungkang longhouse. The area was surrounded by *Eugeissona insignis*, a flowering plant in the palm family which produces fruits that macaques feed on, according to the local communities. Located between site B4 and the longhouse was an approximately 5-m long stream which most villagers had to cross to get to their farms. Site SM, approximately 1 km east of Bungkang, was situated near Sungai Malaban, a slow-moving stream. This site was a favourite hunting area for the villagers and long-tailed macaques were sighted during one of the early collections. Both sites B4 and SM were secondary forests with little vegetation on the forest ground and were approximately 500 m apart. A total of 22 mosquito collections were undertaken at these two sites (B4: eight late and two early collections; SM: six late and six early collections).

### Mosquito collection, identification and dissection

Prior to each collection, tissue moistened with distilled water was placed on the base of each 50 mm × 19 mm cylindrical specimen tubes (SAMCO, UK) followed by plugging the tube opening with a ball of cotton wool. All mosquitoes were collected using the bare-leg catch method where any mosquitoes found landing on the legs of the collectors were trapped using the specimen tubes. The mosquitoes were brought to the field laboratory for morphological identification where the non-anophelines were identified to their genera while the anophelines were identified to their species/group using taxonomic keys^41,42,55^. Salivary glands of each of the *Anopheles* were dissected and preserved in individual 1.5-mL microcentrifuge tubes (AXYGEN, USA) containing 0.5 mL absolute alcohol. The preserved salivary glands were transported to the main laboratory at Universiti Malaysia Sarawak under room temperature. This study was approved by the Medical Ethics Committee of Universiti Malaysia Sarawak and by the Medical Research and Ethics Committee, Ministry of Health Malaysia (NMRR-10-1194-7854). All field staff and volunteers who carried out mosquito collections were provided with antimalarial prophylaxis.

### DNA extraction and PCR detection of *Plasmodium* species

Absolute alcohol preserving the salivary glands was first dried prior to DNA extraction. Genomic DNA of the dried salivary glands was extracted using DNeasy Blood and Tissue Kit (QIAGEN, Germany) according to the manufacturer's protocol and stored at 4 °C until required. Nested PCR assays to detect *Plasmodium* DNA were initially undertaken and *Plasmodium*-positive samples were then subjected to PCR assays with primers specific for *P. coatneyi, P. cynomolgi, P. fieldi, P. inui* and *P. knowlesi* as described previously^18^.

### Generating *Plasmodium* SSUrRNA and *Anopheles* COI gene amplicons for cloning and sequencing

The SSUrRNA genes were amplified by PCR assays using primers rPLU1 and rPLU5^56^ while amplicons for *Anopheles* COI were generated with primers SCOIF (5′-GGA TTT GGA AAT TGA TTA GTT CCT T-3′) and AnCOX1R (5′-CCT AAA TTT GCT CAT GTT GCC-3′) using the Phusion High-Fidelity DNA Polymerase (THERMO FISHER SCIENTIFIC, USA) as described previously^18^ to produce ~ 1405 bp-long blunt-ended amplicons for blunt-end cloning. Amplicons generated were then cloned and sequenced according to the protocol described by Ang et al.^18^. For the COI inserts, internal primer AnCOX1F (5′-CTA GTG TGC TTC CCA TGG AGA TAG-3′) was used for sequencing.

### Sequence alignment and phylogenetic analysis

Multiple sequences generated from this study and those obtained from GenBank were aligned using the default parameters of ClustalW within the LaserGene 7.1 programme (DNASTAR, USA). Reference sequences obtained from GenBank are listed in the additional file (see Supplementary Tables S2 and S3). The best nucleotide substitution models were calculated using MEGA 7.0.21 and the models with the lowest Bayesian Information Criterion (BIC) score were selected. Subsequently, phylogenetic trees were constructed by the Maximum Likelihood (ML) method using MEGA 7.0.21 with bootstrap values calculated from 1000 replicates^57^.

## Supplementary Information

Supplementary Information.

## Data Availability

The datasets generated during and/or analysed during the current study are available from the corresponding author on reasonable request.
