# Supplementary material for: New vectors that are early feeders for Plasmodium knowlesi and other simian malaria parasites in Sarawak, Malaysian Borneo
Source: Sci Rep. 2021 Apr 8;11:7739. doi: 10.1038/s41598-021-86107-3 (PMC8032675; doi:10.1038/s41598-021-86107-3)
Supplement: Supplementary file 1 — Supplementary Information. [file 41598_2021_86107_MOESM1_ESM.pdf]

New vectors that are early feeders for *Plasmodium knowlesi* and other simian malaria parasites in the Betong Division of Sarawak, Malaysian Borneo.

Joshua Xin De Ang[1], jtopsch@gmail.com

Khatijah Yaman[1], ykhatijah@unimas.my

Khamisah Abdul Kadir[1], akkhamisah@unimas.my

Asmad Matusop[2], asmadmatusop@yahoo.com

Balbir Singh[1*], bsingh@unimas.my

[1]Malaria Research Centre, Faculty of Medicine & Health Sciences, Universiti Malaysia Sarawak, Kuching, Sarawak, Malaysia

[2]Sarawak Department of Health, Kuching, Sarawak, Malaysia

[*]Corresponding author

Supplementary Table S1. GPS coordinates for collection sites.

Longhouse	Site code	GPS coordination
Bungkang	B1	1°26'28"N 111°39'04"E
	B2	1°26'46"N 111°39'11"E
	B3	Could not be obtained
	B4	Could not be obtained
	SM	Could not be obtained
Kampung Pelikoi	KP1	1°35'31"N 111°39'09"E
	KP2	1°35'02"N 111°39'07"E
Nanga Buai	NB1	1°26'15"N 111°37'04"E
Nanga Keron	NK1	1°27'21"N 111°38'03"E
	NK2	1°27'30"N 111°38'11"E
	NK3	1°27'19"N 111°38'15"E
Rantau Layang	RL1	1°33'14"N 111°44'48"E
	RL2	1°32'40"N 111°44'30"E
	RL3	1° 33'9"N 111° 44'13"E

Supplementary Table S2. *Plasmodium* SSUrDNA sequences and their GenBank accession numbers.

Species/Sample ID	Isoform	GenBank Acc No.	References
P. gonderi	S	AB287269	1
	A	AB287270	
	A	AB287271	
P. fragile	S	AB287272	
	A	AB287273	
P. coatneyi	S	AB265789	
	A	AB265791	
P. inui	S	AB287275	
	A	AB287276	
P. hylobati	S	AB287278	
	A	AB287279	
	A	AB287280	
P. fieldi	S	AB287281	
	A	AB287284	
P. simiovale	S	AB287285	
	A	AB287286	
P. cynomolgi	S	AB287288	
	A	AB287289	
P. knowlesi	A	DQ641519	Unpublished
	A	DQ641521	
	S	DQ350255	
	S	DQ350260	
	S	DQ350262	
P. sp	A	FJ619074	
	A	FJ619077	
	A	FJ619087	
P. vivax	A	U83877	Unpublished
	A	U07367	2
	S	U07368	
	A	U03079	3
P. falciparum	A	M19172	4
	S	M19173	
B0870A9	-	MN535325	Present study
B1056A1	-	MN535326	
B1056A5	-	MN535327	
B1056A10	-	MN535328	
B1056A20	-	MN535329	
B1056A23	-	MN535330	
B1056A25	-	MN535331	
B1056A31	-	MN535332	
B1056A35	-	MN535333	
B1056A38	-	MN535334	
B1057A25	-	MN535335	
B1057A33	-	MN535336	
B1388A2	-	MN535337	
B1388A15	-	MN535338	
B1388A18	-	MN535339	
B1388A20	-	MN535340	
B1388A22	-	MN535341	
B1388B1	-	MN535342	
B1388B2	-	MN535343	
B1388B3	-	MN535344	

B1388B8	-	MN535345	
B1444A1	-	MN535346	
B1444B1	-	MN535347	
B1444B2	-	MN535348	
B1991A14	-	MN535351	
B1991A19	-	MN535352	
B2000A4	-	MN535353	
B2000A10	-	MN535354	
B2000A22	-	MN535355	
B2000A37	-	MN535356	
B2000A39	-	MN535357	
B0362A91	-	MN535358	
B0362B34	-	MN535359	
B0362C1	-	MN535360	
B0841B4	-	MN535361	
B0870A7	-	MN535362	
B1056A11	-	MN535363	
B1056A15	-	MN535364	
B1056A17	-	MN535365	
B1056A24	-	MN535366	
B1056A33	-	MN535367	
B1056A36	-	MN535368	
B1056B1	-	MN535369	
B1170A1	-	MN535370	
B1283A23	-	MN535371	
B1283B11	-	MN535372	
B1999A9	-	MN535373	
B1999A10	-	MN535374	
B2000A26	-	MN535375	
B2000A36	-	MN535376	
B2000A40	-	MN535377	
B2000B10	-	MN535378	
B2000B29	-	MN535379	

Supplementary Table S3. CO1 sequences of *Anopheles* mosquitoes and their GenBank accession numbers.

Species Group	Species/Sample ID	GenBank Acc. No	Geographic distribution in Malaysia	Reference
Leucosphyrus	*An. introlatus*	MG002550.1	Hulu Selangor, Selangor	Unpublished
		MG002551.1		
		MG002552.1		
		MG002553.1		
		MG002554.1		
		MG002555.1		
		MG002556.1		
		MG002557.1		
		MG002558.1		
	An. latens	MG002559.1	Tawau, Sabah	
		MG002560.1		
		MG002561.1		
		MG002562.1		
		MG002563.1		
		MG002564.1		
		MG002565.1		
	B0362	MN520354	Betong, Sarawak	Present study
	B1056	MN520355		
	B1057	MN520356		
	B1170	MN520357		
	B1283	MN520358		
	B1444	MN520359		
Umbrosus	*An. letifer*	KF564694	Singapore	[5]
		KF564695		
	B0859	MN520365	Betong, Sarawak	Present study
	B0861	MN520367		
	B0863	MN520368		
	B0864	MN520369		
	B0870	MN520370		
	B0871	MN520371		
	B0877	MN520372		
	B0911	MN520373		
	B0912	MN520374		
	B0916	MN520375		
	B0917	MN520376		
	B1135	MN520377		
	B1286	MN520378		
	B1352	MN520379		
	B1388	MN520380		
	B1446	MN520381		
	B1882	MN520382		
	B1883	MN520383		
	B1902	MN520384		
	B1903	MN520385		
	B1976	MN520386		
	B1980	MN520387		
	B1981	MN520388		
	B1991	MN520389		
	B1996	MN520390		
	B1998	MN520391		
	B1999	MN520392		
	B2000	MN520393		
	B2173	MN520394		

References

1. Nishimoto, Y. *et al.* Evolution and phylogeny of the heterogeneous cytosolic SSU rRNA genes in the genus Plasmodium. *Mol. Phylogenet. Evol.* **47**, 45–53 (2008).
2. Li, J., Wirtz, R. A., McConkey, G. A., Sattabongkot, J. & McCutchan, T. F. Transition of *Plasmodium vivax* ribosome types corresponds to sporozoite differentiation in the mosquito. *Mol. Biochem. Parasitol.* **65**, 283–289 (1994).
3. Qar, S. H., Goldman, I. F., Pieniazek, N. J., Collins, W. E. & Lal, A. A. Blood and sporozoite stage-specific small subunit ribosomal RNA-encoding genes of the human malaria parasite *Plasmodium vivax*. *Gene* **150**, 43–49 (1994).
4. McCutchan, T. F. *et al.* Primary sequences of two small subunit ribosomal RNA genes from *Plasmodium falciparum*. *Mol. Biochem. Parasitol.* **28**, 63–68 (1988).
5. Chan, A. *et al.* DNA barcoding: complementing morphological identification of mosquito species in Singapore. *Parasit. Vectors* **7**, 12 (2014).
